# Supplementary figures and images for: Regression of Lung Cancer in Mice by Intranasal Administration of SARS-CoV-2 Spike S1
Source: Cancers (Basel). 2022 Nov 17;14(22):5648. doi: 10.3390/cancers14225648 (PMC9688283; doi:10.3390/cancers14225648)

**2C****BAD**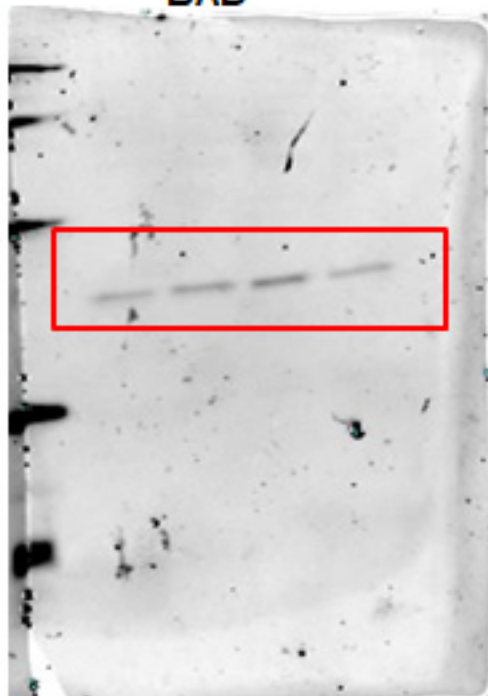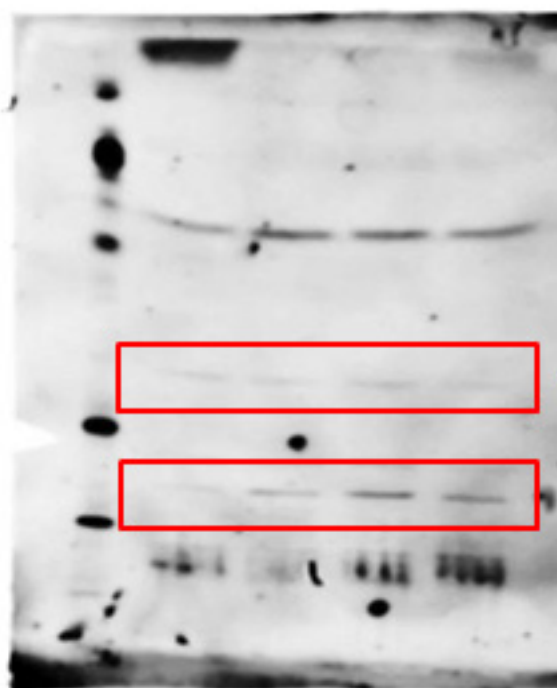**Caspase 3****Cleaved  
caspase 3****Actin**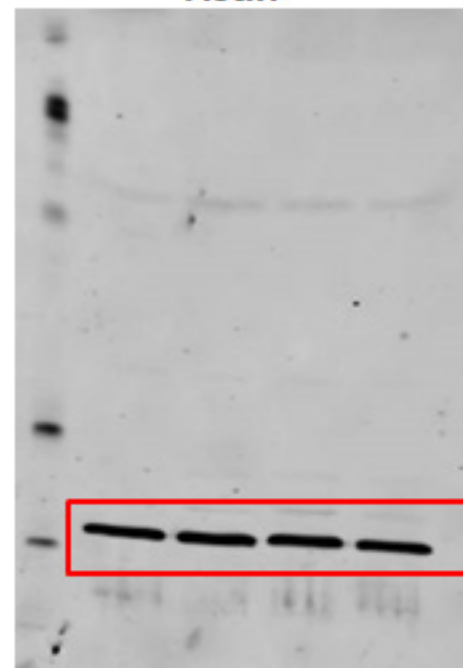**2G****Bcl2**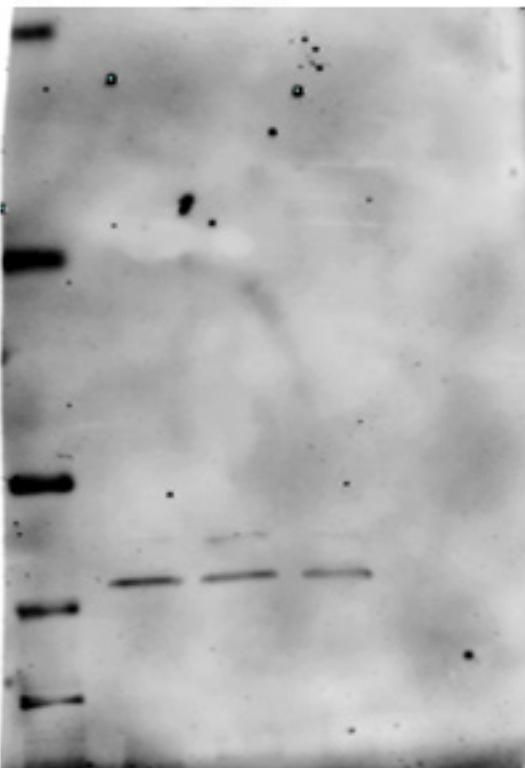**Actin**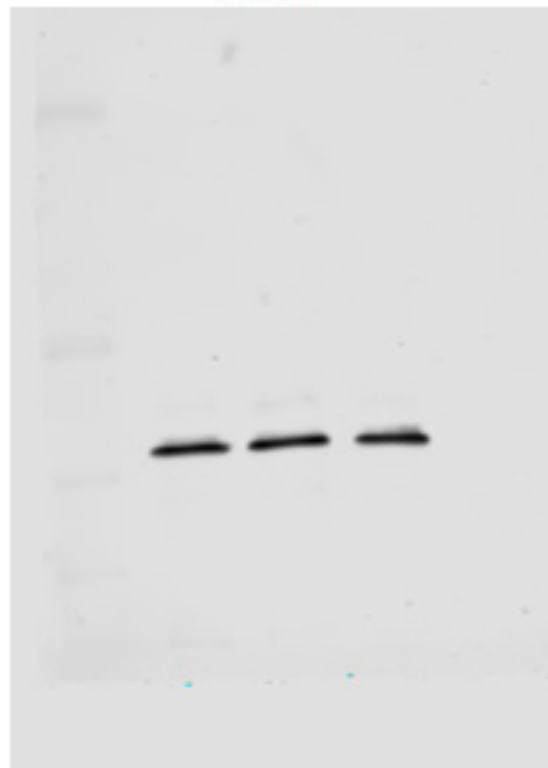

**Figure S1. Raw Western blots for Figure 3C & 3G.**

Supplement: Supplementary file 1 [file cancers-14-05648-s001.zip › cancers-2043594-supplementary.pdf]
